# Intact landscape promotes gene flow and low genetic structuring in the threatened Eastern Massasauga Rattlesnake

**DOI:** 10.1002/ece3.7480

**Published:** 2021-05-02

**Authors:** Nathan Kudla, Eric M. McCluskey, Vijay Lulla, Ralph Grundel, Jennifer A. Moore

**Affiliations:** ^1^ Biology Department Grand Valley State University Allendale MI USA; ^2^ Department of Geography IUPUI Indianapolis, IN USA; ^3^ Great Lakes Science Center U.S. Geological Survey Chesterton IN USA

**Keywords:** dispersal, fragmentation, Island, reptile, Snake, spatial genetics, species distribution modeling

## Abstract

Genetic structuring of wild populations is dependent on environmental, ecological, and life‐history factors. The specific role environmental context plays in genetic structuring is important to conservation practitioners working with rare species across areas with varying degrees of fragmentation. We investigated fine‐scale genetic patterns of the federally threatened Eastern Massasauga Rattlesnake (*Sistrurus catenatus*) on a relatively undisturbed island in northern Michigan, USA. This species often persists in habitat islands throughout much of its distribution due to extensive habitat loss and distance‐limited dispersal. We found that the entire island population exhibited weak genetic structuring with spatially segregated variation in effective migration and genetic diversity. The low level of genetic structuring contrasts with previous studies in the southern part of the species’ range at comparable fine scales (~7 km), in which much higher levels of structuring were documented. The island population's genetic structuring more closely resembles that of populations from Ontario, Canada, that occupy similarly intact habitats. Intrapopulation variation in effective migration and genetic diversity likely corresponds to the presence of large inland lakes acting as barriers and more human activity in the southern portion of the island. The observed genetic structuring in this intact landscape suggests that the Eastern Massasauga is capable of sufficient interpatch movements to reduce overall genetic structuring and colonize new habitats. Landscape mosaics with multiple habitat patches and localized barriers (e.g., large water bodies or roads) will promote gene flow and natural colonization for this declining species.

## Graphical abstract

We investigated fine‐scale genetic patterns of the federally threatened Eastern Massasauga Rattlesnake (*Sistrurus catenatus*) on a relatively undisturbed island in northern Michigan, USA. We detected an isolation‐by‐distance pattern and weak genetic structuring with spatially segregated variation in effective migration and genetic diversity. The lack of strong genetic structuring in this intact landscape suggests that the Eastern Massasauga is capable of sufficient interpatch movements to colonize new habitats and maintain gene flow at this scale.

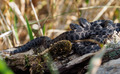

## 1 INTRODUCTION

Genetic structuring of populations is shaped by aspects of habitat including quantity, configuration, quality, barriers, and structural connectivity (Locher et al., 2015; Miller et al., 2018; Nevill et al., 2019), by species’ characteristics including behavior, dispersal, and migration (Bryja et al., 2009; Milot et al., 2008); by population density (Moore et al., 2014); and by other factors affecting isolation and movement (Powney et al., 2011). This amalgam of environment, ecology, and life‐history characteristics affects gene flow among populations at multiple geographic scales, thus defining the functional connectivity of populations across landscapes. Even for a single species, patterns of gene flow can vary between locales, resulting, for example, in different levels of genetic structuring between populations (Coulon et al., 2010; Dudaniec et al., 2012; Jørgensen et al., 2005). We can assume that in many circumstances the historic landscapes in which a species resides have been differentially modified following European colonization, with some sites retaining more of their historic character (Caplat et al., 2016; Landguth et al., 2010; Vera‐Escalona et al., 2015). Understanding the factors that influence functional connectivity among sites that retain more or less of their historic character can help us maintain historic levels of gene flow by guiding habitat management efforts for a species.

Different environmental contexts may produce varying degrees of genetic structure, such as an undisturbed site compared to a fragmented landscape (Moore et al., 2011). Although fragmentation can initially increase population densities due to crowding in remnant patches, over time populations tend to decline due to reduced habitat availability, increased edge effects, and limited migration (Fletcher et al., 2018; Haddad et al., 2017), thereby hastening differentiation among demes (Delaney et al., 2010; Yamamoto et al., 2019). The reduction of genetic variability in small, isolated populations, due to drift and lack of gene flow, can limit their adaptive potential (Frankham, 1995; Wade et al., 2017) and increase inbreeding depression, making them vulnerable to environmental and demographic stochasticity (Fagan & Holmes, 2006; Soulé et al., 1986). These effects are more prominent for less mobile species (e.g., Amos et al., 2014), thus enhancing structural connectivity may be particularly important for gene flow of less mobile organisms within fragmented landscapes; otherwise, more direct forms of intervention may be necessary (Frankham, 2015).

**FIGURE 1:**
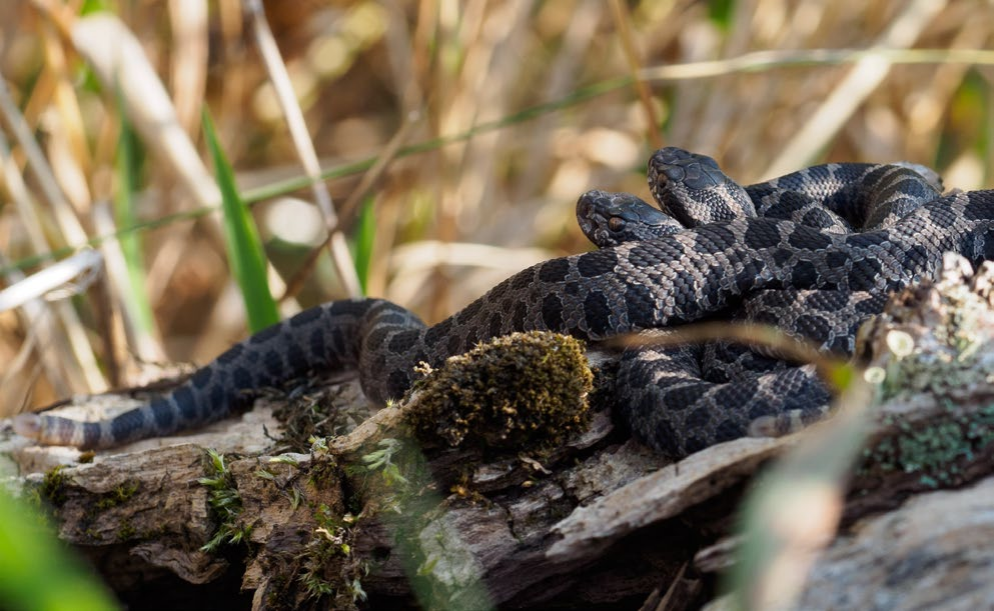
Neonate Eastern Massasauga Rattlesnakes found basking together at a sampling site on Bois Blanc Island, Michigan (photo credit: E. McCluskey)

Throughout most of its Great Lakes distribution in North America spanning from Iowa to New York, the Eastern Massasauga (*Sistrurus catenatus*; Figure 1) exemplifies the ramifications of population isolation for a dispersal‐limited species. Having variable, but typically small, home range sizes (25 ha (Weatherhead & Prior, 1992), 4.02 ha (Marshall et al., 2006), 1.3 ha (Moore & Gillingham, 2006), and 0.98 ha (Reinert & Kodrich, 1982)) within their wetland‐associated grassland habitats has enabled Eastern Massasaugas to persist in heavily fragmented environments, particularly in the southern part of its range (Iowa, Illinois, Indiana, Ohio, and Pennsylvania), but not without genetic consequences (see below). However, similar to other ambush predators (Shine & Fitzgerald, 1996; Webb & Shine, 1998), and given their small size relative to most rattlesnake species, Eastern Massasaugas exhibit limited dispersal and movement (maximum range lengths 1–2 km; DeGregorio et al., 2011; Durbian et al., 2008) that reduces their ability to colonize new habitats (McCluskey et al., 2018).

Eastern Massasaugas are listed as Threatened in the United States under the Endangered Species Act and in Canada under the Species at Risk Act; therefore, concerns about inbreeding, functional connectivity, and population viability have prompted several genetic investigations across the species’ range (Chiucchi & Gibbs, 2010; DiLeo et al., 2013; Martin et al., 2021; Sovic et al., 2019). The genetic consequences of population isolation and subsequent reduction or cessation of gene flow are evidenced by low effective population sizes (<50 individuals), estimated for multiple populations throughout the range, and an expected reduction of existing population genetic variation of at least 20% in the next 100 years (Baker et al., 2018; Bradke et al., 2018; Martin et al., 2021; Sovic et al., 2019).

In regional analyses of Eastern Massasauga genetic structure from Illinois, Ohio, and Pennsylvania, Chiucchi and Gibbs (2010) found highly structured populations, even when populations were separated by relatively small distances (<7–25 km). Sovic et al. (2019) re‐evaluated the Chiucchi and Gibbs (2010) data and confirmed that isolation coupled with small population size caused declines in genetic diversity for many of these populations. These results suggest a populations‐as‐islands model of conservation management for the Eastern Massasauga in the highly fragmented portion of its range. Conversely, DiLeo et al. (2013) showed that genetic isolation is not ubiquitous for this species in their study of Eastern Massasauga populations along eastern Lake Huron (Bruce Peninsula and East Georgian Bay) in Ontario. They identified multiple population clusters along East Georgian Bay but cluster membership for two of these extended beyond 25 km, exceeding the scale of the structured regional populations from Chiucchi and Gibbs (2010). Bruce Peninsula followed a similar pattern, clustering as a single population despite being broadly sampled (~40 km).

Contrasting descriptions of levels of genetic population structure from different parts of this species range illustrate the importance of environmental context regarding intensity of land modification and habitat fragmentation when predicting the degree of population genetic structuring. The results from DiLeo et al. (2013) imply that limited dispersal capabilities documented for the Eastern Massasauga (Weatherhead & Prior, 1992) do not always equate to highly structured populations even at relatively broad scales. However, for a dispersal‐limited species to have low genetic structure likely requires spatially cohesive and extensive habitat with few barriers (Caizergues et al., 2003; Gibbs, 1998). The Ontario populations described by DiLeo et al. (2013) are in remote areas with minimal development or barriers (though Bruce Peninsula has substantial tourism traffic and an associated road network; Reed & McKenzie, 2010); therefore, Eastern Massasauga movement should be less restricted on Bruce Peninsula compared to the fragmented landscapes sampled by Chiucchi and Gibbs (2010) for their regional analyses.

The question remains as to whether population isolation and strong genetic structuring are the norm for Eastern Massasaugas, or whether relatively intact landscapes do broadly promote connectivity. If the latter, targeted habitat management in fragmented landscapes would have the potential to restore functional connectivity and enhance population viability. Here, we determine whether gene flow and low genetic structuring are present elsewhere in the Eastern Massasauga's range. Our study site, Bois Blanc Island (BBI), is located in Lake Huron, between Michigan's Upper and Lower Peninsulas. BBI resembles the part of Ontario with the highest abundance of Eastern Massasaugas, with numerous occupied discrete habitat patches, minimal development aside from roads, and is situated at the species’ northern distribution limit (Szymanski et al., 2016). Our spatial sampling is comparable to the fine‐scale structuring observed in Chiucchi and Gibbs (2010) (~7 km), but much finer than the broader genetic clusters identified by DiLeo et al. (2013) in relatively intact Ontario landscapes (25–40 km). The broad scale analysis by DiLeo et al. (2013) was vital for demonstrating the capacity of Eastern Massasauga to maintain gene flow over larger areas but did not evaluate spatial genetic patterns pertaining to structuring at a scale representative of interpatch movement for this species. Therefore, our study aimed to fill this gap in knowledge and represents the first effort to examine fine‐scale spatial genetic structure for Eastern Massasaugas inhabiting a landscape with high patch occupancy and abundance.

Specifically, we aimed to (a) quantify available habitat and landscape connectivity across this comparatively unmodified landscape and (b) assess spatial genetic relationships using approaches not employed in previous Eastern Massasauga studies, yet at a spatial scale where strong genetic structuring has been found in Illinois, Ohio, and Pennsylvania (Chiucchi & Gibbs, 2010). We incorporated species distribution modeling (SDM) to reveal the extent and connectedness of habitat on the island so our genetic results could be evaluated within the context of habitat availability. An unmodified landscape by itself may not be sufficient for promoting successful gene flow in a dispersal‐limited species without a robust habitat network. Given the similar environmental contexts (i.e., low human population density, contiguous tracts of natural vegetation), we predicted Eastern Massasauga populations on BBI would exhibit limited genetic structuring, similar to the Lake Huron region, Ontario populations. Developing Eastern Massasauga management strategies that create or maintain connectivity to promote migration and natural colonization is a priority. Our study assessed genetic processes within a landscape with high Eastern Massasauga abundance, low human development, and lacking active management, thus offering insights into Eastern Massasauga dispersal patterns in landscapes with minimal human intervention.

## 2 METHODS

### 2.1 Study area

**FIGURE 2:**
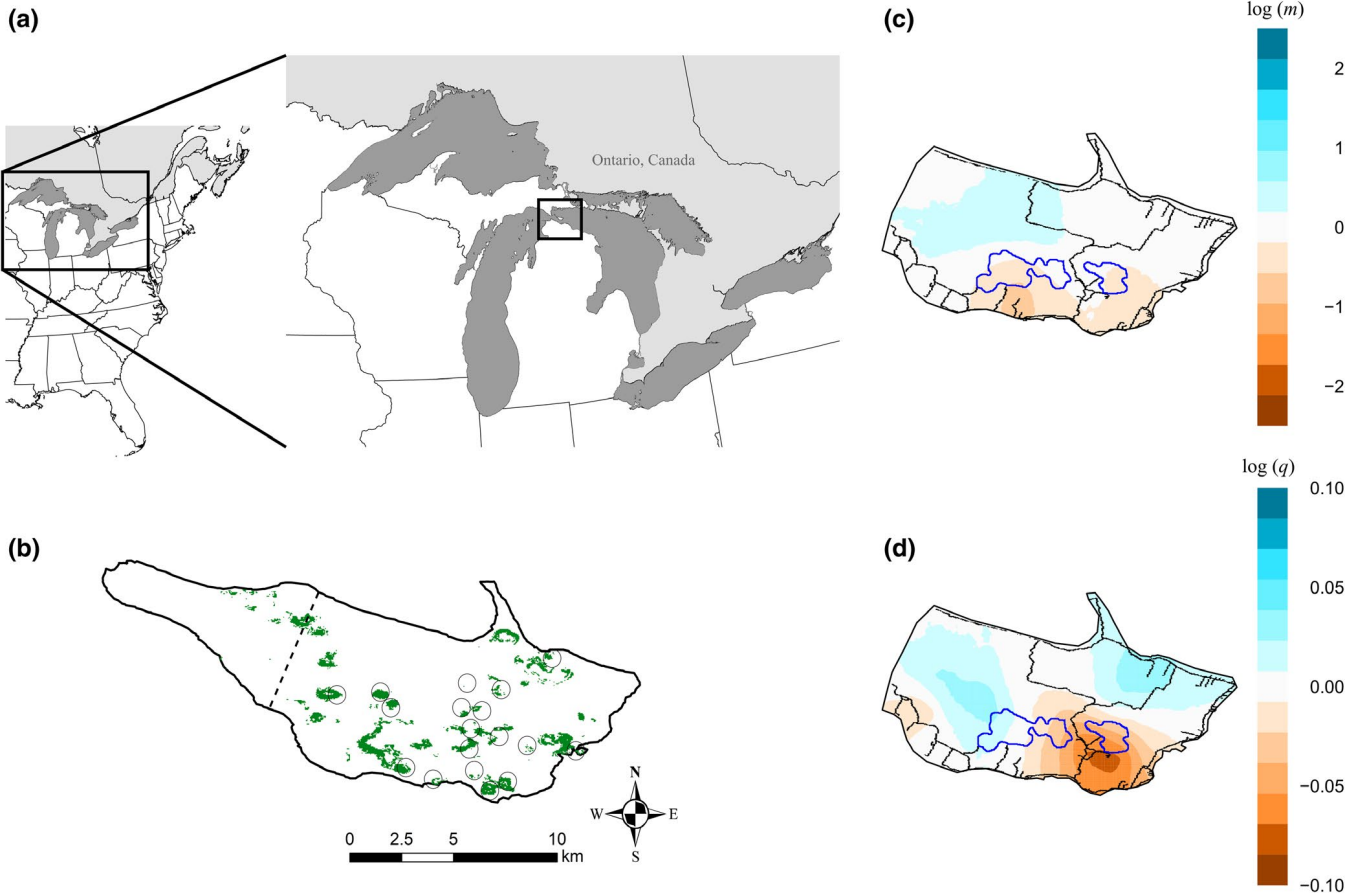
(a) Location of Bois Blanc Island (45°46'30"N 84°28'44"W) in Lake Huron between the Upper and Lower Peninsulas of Michigan. (b) Map of Bois Blanc Island with circles indicating sampling areas (exact location adjusted to deter illegal collection) and habitat patches (>1 ha) from the species distribution model. The habitat and estimated effective migration surfaces (EEMS) analyses were based on the portion of the island southeast of the dashed line, reflected in parts c and d. (c) Effective migration based on genetic dissimilarities between demes with blue areas indicating more migration than expected under isolation by distance and orange/brown showing reduced migration. (d) Effective diversity based on genetic dissimilarities between individuals from the same deme showing areas of higher genetic diversity in blue and lower genetic diversity in orange/brown. Both c and d show the two inland lakes outlined in blue and the island road network in black

Bois Blanc Island (BBI) is one of the larger islands (88 km^2^) in the Great Lakes, situated in Lake Huron between the Upper and Lower Peninsulas of Michigan, USA. (Figure 2a). Predominantly forested with wetlands and beaver meadows interspersed throughout, the island is largely undeveloped with a small resident human population (95 people; 0.75 people km^−2^ (U.S. Census Bureau, 2010). The island is estimated to have been deglaciated 11,200–11,000 years ago (Larsen, 1987), after which BBI was isolated from the Upper and Lower Peninsula until 10,300 years ago when lake levels dropped, connecting BBI to the Lower Peninsula (Larsen, 1987). This period is likely when Eastern Massasaugas colonized BBI. The water level of the lakes once again rose and BBI became isolated around 4,500–4,000 years ago (Larsen, 1987). The island was logged from 1900 to 1930 using three narrow gauge trains that ran through the interior of the island transporting logs, which may have provided additional open‐canopy habitat for Eastern Massasaugas (Sanborn et al., 2012).

### 2.2 Sampling methods

We conducted visual encounter surveys from 24–26 July in 2015, 26–31 May and 8–10 September in 2017, and 27 May–3 June in 2018. Survey sites were located using Eastern Massasauga locations previously documented by the Michigan Natural Features Inventory (MNFI; https://mnfi.anr.msu.edu/) in 1978, 1979, 1990, and 2010 and by identifying open‐canopy areas using aerial imagery (ArcMap Basemap; Google Earth). We distributed survey sites to ensure that the heterogeneity of the landscape and genetic variation of individuals was adequately sampled (Figure 2b; Balkenhol et al., 2015). At the location of each snake capture, we recorded soil temperature, shaded air temperature, and cloud cover. We measured each individual's weight and length and probed the cloaca and palpated snakes to determine sex and reproductive status. Individual snakes were marked with a subdermal passive integrated transponder (PIT) tag. All snakes were scanned prior to processing for the presence of a PIT tag, to avoid resampling the same individual. Up to 200 µl of blood was collected from the caudal artery of each individual and stored in 95% ethanol. After completing data collection, snakes were returned to their capture site and released. All equipment that contacted a snake was either sterilized with a 10% bleach solution or single‐use equipment was changed out between individuals, to prevent the spread of snake fungal disease (*Ophidiomyces ophiodiicola*; Allender et al., 2016; Rzadkowska et al., 2016).

### 2.3 Laboratory analyses

We extracted DNA from blood samples of 102 unique Eastern Massasaugas using QIAGEN DNeasy Tissue kits following the manufacturer's protocol. We genotyped individuals at 16 microsatellite loci developed by Anderson et al. (2010). Each 10 µl PCR reaction contained 20–100 ng DNA, 10 mM Tris–HCl, 50 mM KCl, 1.5 mM MgCl_2_, 1 µl of 0.5 mg/ml bovine serum albumin, 0.2 µl deoxynucleotide solution mix (0.2 mM of each), 1 U *Taq* DNA polymerase, 0.6 µl primers (0.3 µM of each, with fluorescently labeled forward primer), and 5 µl double‐distilled H_2_O. We amplified microsatellite DNA loci with an Eppendorf Mastercycler nexus gradient thermal cycler and followed the protocol established by Anderson et al. (2010), except for modifying annealing temperatures. Loci with their modified annealing temperatures are as follows: *Scu215* (50°C), *Scu210* (56°C), *Scu211* (56°C), *Scu212* (56°C), *Scu213* (56°C), *Scu214* (56°C), *Scu216* (56°C), *Scu202* (60°C), *Scu203* (60°C), *Scu205* (60°C), *Scu200* (62°C), *Scu201* (62°C), *Scu204* (62°C), *Scu206* (62°C), *Scu208* (62°C), and *Scu209* (62°C). To detect any potential contamination of our PCR runs, we used a negative control for each reaction set. Following PCR amplification, fragments were analyzed on an ABI3130xl Genetic Analyzer (Applied Biosystems, Foster City, CA, USA), at Annis Water Resources Institute, Grand Valley State University. We manually scored fragments using PeakScanner (vers. 2.0). Of the 102 samples, we reamplified and genotyped ~10% (11 individuals) of our total sample to verify our genotyping results and to calculate a PCR and allele scoring error rate.

### 2.4 Genetic analyses

We tested all samples for departures from Hardy–Weinberg equilibrium and linkage disequilibrium using exact tests in the program GENEPOP (vers. 4.2) (Rousset, 2008). Using the program GenAlEx (vers. 6.5) (Peakall & Smouse, 2005), we calculated the number of alleles, effective number of alleles, observed heterozygosity, and expected heterozygosity. When investigating spatial genetic patterns, it is important to interpret results in light of the isolation‐by‐distance (IBD) pattern that is present (Perez et al., 2018). To assess patterns of isolation by distance (IBD), we used Mantel tests within the R (vers. 3.6.1) package ADEGENET (Jombart, 2008).

We tested for spatial genetic patterns using spatial principal component analysis (sPCA) implemented in ADEGENET (Jombart, 2008). e chose the Gabriel graph option for the spatial network as it represents a compromise between Delaunay triangulation that includes more connections than is likely for Eastern Massasauga dispersal and overly restrictive nearest neighbor options (Teich et al., 2014). Unlike Bayesian clustering programs such as STRUCTURE (Falush et al., 2003; Pritchard et al., 2000), sPCA does not assume Hardy–Weinberg equilibrium and linkage equilibrium. sPCA uses Moran's *I* to identify patterns of spatial autocorrelation and is effective at detecting cryptic genetic structure (Jombart, 2008; Schwartz & McKelvey, 2008; Vergara et al., 2015). The sPCA components can reveal both global (positive eigenvalues) and local (negative eigenvalues) structures. When global structure is significant, it can indicate either clusters or clines in the dataset compared to between‐individual genetic differences reflected by the local scores. We assessed both patterns with the “spca_randtest” function recommended to increase statistical power (Montano & Jombart, 2017) using 9,999 permutations.

We used two methods to identify the most likely number of genetic clusters among samples on BBI, first with the Bayesian clustering program STRUCTURE (vers. 3.4.1) (Pritchard et al., 2000). Our STRUCTURE analysis included 20 independent runs for K = 1 through K = 15 (approximate number of sampling areas) with an initial burn‐in of 50,000 followed by 500,000 iterations under the admixture model without assigned population information. We determined the most likely number of clusters using web‐based StructureSelector (Li & Liu, 2018) which provides the selected K from the four estimators (MedMeaK, MaxMeaK, MedMedK, MaxMedK) proposed by Puechmaille (2016) and used the methods of Evanno et al. (2005). Puechmaille (2016) demonstrated that the Evanno method can underestimate the number of genetic clusters when there is uneven sampling. We collected 1–13 samples per site. Sampling was often spread across adjacent habitat complexes making it difficult to define population clusters based on sampling location; however, StructureSelector recommends such designations be made for the Puechmaille (2016) method so seven general groups were assigned based on sampling location for this analysis (Figure A1). We also used a multivariate method, discriminant analysis of principal components (DAPC) implemented in the R package ADEGENET (Jombart, 2008). Like sPCA, DAPC does not rely on traditional population genetic assumptions. DAPC is effective at discerning genetic clustering in more complex population genetics models (Jombart et al., 2010).

We also evaluated spatial genetic patterns on BBI with EEMS (estimated effective migration surfaces). EEMS is a visualization tool, developed for use in systems where IBD is present but deviations due to barriers or corridors have also contributed to rates of historical gene flow. Effective migration rate is based on a stepping‐stone model under idealized settings that would generate the same genetic dissimilarities between demes as seen in the data (Petkova et al., 2016). The effective diversity rate assesses genetic dissimilarities that exist between individuals within a single deme (Petkova et al., 2016). The EEMS program maps effective migration surfaces where genetic similarity decays more quickly than expected under IBD, indicating reduced gene flow (Petkova et al., 2016; Silliman, 2019). Similarly, effective diversity surfaces indicate regions where genetic diversity is higher or lower than average. We calculated descriptive statistics for the high and low genetic diversity regions from this analysis using the R packages STRATAG (Archer et al., 2017) and PopGenReport (Adamack & Gruber, 2014). Our EEMS analysis was focused on the eastern two‐thirds of the island. We first adjusted most default parameters so acceptance rates fell in the recommended 10%–40% range. We left the negBiProb parameter at the default value to maintain more tiles for each run. Then, we ran three independent chains for 40, 60, 80, and 100 demes for 2,000,000 MCMC iterations with 1,000,000 iterations of burn‐in, thinning every 10,000 iterations followed by visualization with the included rEEMSplot R plotting package (https://github.com/dipetkov/eems).

We tested for spatial autocorrelation among the 102 samples to identify patterns of fine‐scale genetic structuring. Using the program GenAlEx, we used a pairwise matrix of genetic and geographic distance to calculate a correlation coefficient (r) for each distance bin. Distance bins were set at 0.5 km and extended from 0 to 5 km. Significant spatial autocorrelation occurred when r was greater or less than the 95% confidence intervals (Peakall et al., 2003).

### 2.5 Habitat analyses

We developed a BBI‐specific SDM for habitat availability and connectivity estimates to complement our genetic results. Our SDM was based on an ensemble of small models (ESM) approach implemented in the R package ecospat (Breiner et al., 2015; Cola et al., 2017) that was developed for situations where few occurrences exist. ESMs consist of independent models built with each pair of variables before creating an ensemble of all models with user‐specific thresholds or weighting options. We created our occurrence dataset (*n* = 28) by spatially thinning all BBI Eastern Massasauga records obtained from the Michigan Natural Heritage Database (1978, 1979, 1990, and 2010) maintained by MNFI and our surveys using a minimum distance of 200 m to avoid overemphasizing environmental data from any individual site. Background point selection was limited to a 10 km buffered region around all occurrence data (clipped to the extent of BBI). We screened a range of candidate environmental predictor variables representing land cover, elevation, and hydrology features potentially important for Eastern Massasauga presence (Supplement 1). First, we used the “corSelect” function from the fuzzySim r package (Barbosa, 2015) to remove highly correlated variables (>0.75), retaining the higher scoring variable from each correlated pair. We further reduced variables using the jackknife of variable importance and training gain from Maxent. These variables were retained for the ESMs: 1 km topographic position index, distance to high wetland potential (merged classes (5–8) from Coastal Change Analysis Program (C‐CAP) wetland potential 2017 (NOAA)), percentage of emergent wetlands (Michigan C‐CAP land cover 2016 (NOAA)) within 300 m, canopy cover (Coulston et al., 2013; Yang et al., 2018) represented by four equal intervals, standard deviation of canopy cover (Coulston et al., 2013; Yang et al., 2018) in a 200 m moving window, and percentage of all wetlands (Michigan Wetlands Map 2018 (USFWS)) within 300 m. Web links to data sources are provided in the supplementary table (Supplement 1).

We generated ESMs using Maxent (Phillips et al., 2006; Phillips & Dudík, 2008) and artificial neural network (ANN) model types separately, then an ensemble combining both. Both Maxent and ANN have been shown to work well in an ESM framework (Breiner et al., 2018). We applied internal ecospat tuning for both Maxent and ANN. All models (Maxent, ANN, ensemble) were run 10 times with fivefold cross‐validation and weighted using Somers’ D (Breiner et al., 2015). We evaluated the three ESM model types with the Boyce Index (Hirzel et al., 2006) that was calculated using the “ecospat.boyce” function for the continuous output of each model within the 10 km background extent. We also compared the three ESM models to three broader SDMs created with the northern and statewide Michigan Eastern Massasauga occurrence data (including some BBI occurrences) to determine the best fit for the BBI data. These comparisons were also based on Boyce Index values calculated within the BBI model background extent.

We applied the threshold that maximized the true skill statistic (TSS) (equivalent to maximizing the sum of sensitivity and specificity; Liu et al., 2013) to our continuous SDM output from the top model to identify patches of suitable habitat on BBI. The smallest habitat patches where we sampled Eastern Massasaugas were between 1–1.5 ha, so we used 1.0 ha as the minimum patch size for our analysis. Using FRAGSTATS (vers. 4.2, McGarigal et al., 2012), we calculated the following for Eastern Massasauga habitat patches on the eastern section of the island, as in the EEMS analysis: percentage of land area (PLAND), clumpiness, a measure of habitat aggregation, and mean Euclidean nearest neighbor (ENN). Clumpiness estimates level of fragmentation but is not strongly influenced by habitat abundance (Wang et al., 2014). Mean ENN is more sensitive to habitat abundance making it less useful for comparing among different landscapes but still informative for understanding dispersal patterns among habitat patches on BBI (Wang et al., 2014).

## 3 RESULTS

### 3.1 Genetic analyses

Across our 102 samples, *Scu209* was removed from our analyses because it was monomorphic. Using the 15 remaining loci, we calculated an allele scoring error rate of 2.98%. After simple Bonferroni correction, all loci were in Hardy–Weinberg equilibrium, while 8 of 105 pairs of loci showed significant linkage disequilibrium. The number of alleles per locus ranged from 2 (*Scu206*) to 11 (*Scu211*; *Scu216*) (Table 1). The average number of alleles across loci was 5.87, and the number of effective alleles was 3.0. Observed heterozygosity across loci ranged from 0.17 (*Scu208*) to 0.79 (*Scu215*) (Table 1). Overall, the average observed heterozygosity for BBI was 0.58 while the expected heterozygosity was 0.60 (Table 1).

**TABLE 1:** Genetic diversity statistics for the 102 Eastern Massasauga sampled on Bois Blanc Island organized by microsatellite locus including number of alleles per locus (N_a_), effective number of alleles (N_e_), observed heterozygosity (H_o_), and expected heterozygosity (H_e_). The microsatellite locus *Scu209* was monomorphic (N_a_ = 1) and excluded from the analysis

Locus	N_a_	N_e_	H_o_	H_e_
*Scu201*	4	1.63	0.41	0.389
*Scu202*	7	2.91	0.66	0.660
*Scu203*	3	2.56	0.61	0.612
*Scu204*	4	3.29	0.60	0.699
*Scu205*	9	3.85	0.75	0.746
*Scu206*	2	1.54	0.33	0.351
*Scu208*	4	1.18	0.17	0.155
*Scu210*	7	4.36	0.78	0.774
*Scu211*	11	5.17	0.76	0.811
*Scu212*	5	2.63	0.62	0.623
*Scu213*	5	2.59	0.55	0.618
*Scu214*	4	1.39	0.26	0.282
*Scu215*	5	4.10	0.79	0.760
*Scu216*	11	5.10	0.75	0.808
*Scu217*	7	2.80	0.63	0.646
**Average**	**5.87**	**3.01**	**0.58**	**0.60**

**FIGURE 3:**
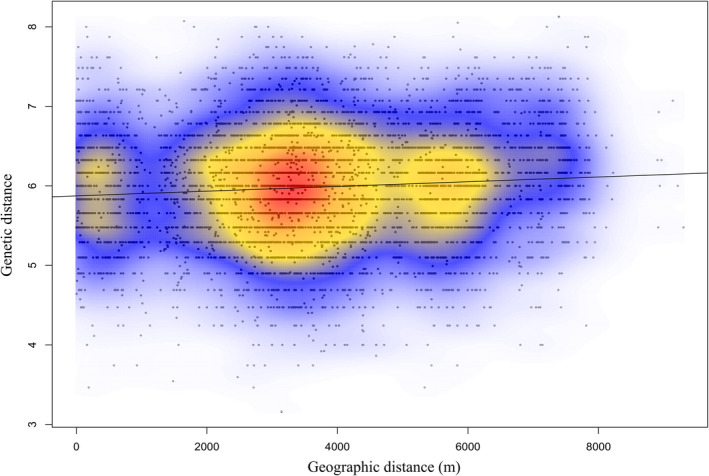
Scatterplot with linear regression line between both distance measures showing the matrix of pairwise genetic distances (y‐axis) using a modified Nei's distance described in Huff et al. (1993) and matrix of pairwise geographic distances (x‐axis) for Eastern Massasauga sampled across Bois Blanc Island (BBI). Warmer colors (yellow, red) within the kernel density indicate higher densities of points. A single dense cluster with most points favors a clinal pattern existing on BBI

We detected significant IBD for Eastern Massasaugas on BBI (Mantel *r* = 0.08). The IBD results are more characteristic of a clinal pattern than defined clusters (Figure 3). Tests for both local and global structure, using sPCA, were nonsignificant (Figure A2). Genetic partitioning is not evident among the BBI sampling areas.

The Evanno method and DAPC selected two and three clusters, respectively. Estimates of MedMeaK, MedMedK, MaxMeaK, and MaxMedK (Puechmaille, 2016) were similar to these results, identifying K = 2 (MedMeaK; MedMedK) and 3 (MaxMeaK; MaxMedK) as the best fit for the data, with cluster membership displaying no clear spatial pattern, consistent with weak structuring and isolation by distance (Figures [Link], [Link], [Link], [Link]). The cluster from the southeast corner of the island exhibited the most consistent assignment of individuals to one cluster.

EEMS showed that effective migration and diversity exhibited similar geographic patterns of high and low areas relative to the mean within our study area on BBI (Figure 2c,d). Effective migration exceeds what is expected under strict IBD in the northwest, and elevated genetic diversity is found in two separate sections of northern BBI. Reduced migration and diversity are concentrated in the southeastern part of BBI, between the two inland lakes and the Lake Huron shoreline (Figure 2c,d). Genetic diversity estimates from the high‐ and low‐diversity zones (Figure 2) showed higher observed and expected heterozygosity in the high diversity areas (Tables A1–A3). There was slightly higher allelic diversity overall in the low‐diversity region though among more individuals (*n* = 46) than the western (*n* = 19) and eastern (*n* = 11) high diversity zones.

**FIGURE 4:**
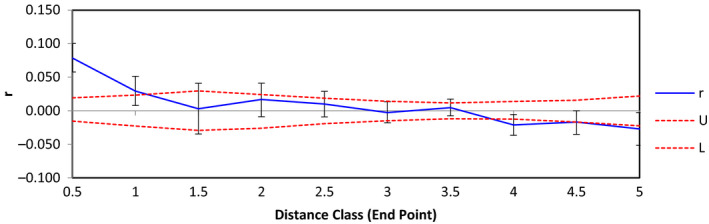
Correlogram plot of Eastern Massasauga samples collected from Bois Blanc Island, Michigan. On the y‐axis, r represents the genetic correlation coefficient while the x‐axis represents the distance class (km) of our samples. The dashed red lines represent 95% upper and lower confidence intervals for the null hypothesis of no spatial genetic structure. Error bars are bootstrapped 95% confidence intervals within each distance class

We identified significant positive spatial autocorrelation between individuals at approximately 1 km on BBI, suggesting Eastern Massasaugas show restricted dispersal within 1 km (Figure 4).

### 3.2 Habitat analysis

Maxent models performed slightly better than ANN and the ANN/Maxent ensemble across the 10 replicate runs (Table A4a–c). However, among the final Somers' D weighted average models, the ANN ESM model was the best model based on Boyce Index (0.98) followed by the ensemble (0.968) and Maxent (0.962). All ESM models outperformed the statewide (ANN = 0.774; Maxent = 0.881) and northern (Maxent = 0.889) broad scale models. After thresholding the ANN ESM model into suitable and unsuitable habitat, 7.2% of the assessed area was considered suitable (Figure 2b). Habitat patches were aggregated as measured by the clumpiness metric (0.82) and close together based on mean Euclidean nearest neighbor (245 m *SD* 183).

## 4 DISCUSSION

Our results confirm that gene flow is possible across Eastern Massasauga populations, even at relatively broad scales (~7 km). Our well‐connected landscape with abundant suitable habitat facilitated sufficient gene flow to result in very limited genetic structure, which is not typical for this species elsewhere (but see DiLeo et al., 2013). Our study took place on an island, but our genetic diversity measures are on par with previous estimates from highly structured populations throughout the range. We show the importance of considering landscape context when inferring spatial genetic patterns, even for a species whose low vagility and movement patterns predict strong genetic structure.

### 4.1 Genetic structuring

Low observed genetic structuring across our island landscape is further evidence that the expansive Ontario genetic clusters (DiLeo et al., 2013) are not an anomaly. Instead, the preponderance of highly structured Eastern Massasauga populations throughout the range may have resulted from anthropogenic modifications of landscapes in which Eastern Massasaugas reside. Our findings on BBI differ from those of Chiucchi and Gibbs (2010) and Sovic et al. (2019) who found high levels of genetic structuring across the range. In their fine‐scale regional analysis, Chiucchi and Gibbs (2010) found strong, significant genetic structuring between pairs of sites in Illinois, Ohio, and Pennsylvania at distances equivalent to, or shorter than, the extent of our BBI study area. This discrepancy of results between our analysis and Chiucchi and Gibbs’ (2010) sites can likely be attributed to differences in habitat quality, quantity, and connectivity. The landscapes surrounding the Chiucchi and Gibbs (2010) regional sites are heavily fragmented by agriculture and roads, thereby limiting overall habitat availability and restricting migration between occupied patches compared to BBI with abundant occupied habitat patches that are well aggregated and in close enough proximity (mean distance < 300 m) to facilitate interpatch movement.

The low levels of structuring found in northern populations (northern Michigan, Ontario) compared to the Eastern Massasauga's southern distribution (Illinois, Ohio, Pennsylvania) likely has more to do with the intensity of anthropogenic landscape modification that transpired across the southern portion of the Eastern Massasauga's range than geographically stratified population structuring (Brown et al., 2005; Byun et al., 2018; Steyaert & Knox, 2008; Yu & Lu, 2018). The Great Lakes region has lost over 50% of its wetland habitat since European colonization but those losses have not been evenly distributed: Illinois (90% lost), Indiana (87%), Ohio (90%), Michigan (50%), and southern Ontario (90%) (Dahl, 1990; Snell, 1987; Suloway & Hubbell, 1994). The individual state/province estimates provide important context for evaluating connectivity among contemporary Eastern Massasauga populations. The substantial wetland losses recorded across the southern portion of the Eastern Massasauga's range likely contributed to the high levels of structuring reported in Chiucchi and Gibbs (2010). Eastern Foxsnake (*Pantherophis gloydi*), a marsh and prairie specialist that is sympatric with the Eastern Massasauga's historical distribution through southern Ontario, northern Ohio, and southeastern Michigan, has shown similar genetic structuring patterns to those reported by Chiucchi and Gibbs (2010) reflecting heavily fragmented areas created by agriculture (Row et al., 2010). In contrast, less agriculture and lower human population densities along the northern extent of the Eastern Massasauga's range have left more contiguous habitat that contributes to the low structuring observed on BBI and parts of Ontario. Historically, extensive prairie habitats through Ohio, Indiana, and Illinois would have supported large Eastern Massasauga populations where low structuring would be expected, as has been observed in prairie populations of Yellow Belly Racers (*Coluber constrictor flaviventris*) and a sister taxon, Western Massasauga (*S. tergeminus*) in Kansas (Klug et al., 2011; McCluskey & Bender, 2014). Alternatively, geographically stratified population structuring could result if northern populations associated with postglacial expansion were less structured than the southern part of the range that was historically differentiated. However, the lack of broadly distributed populations across the southern part of the range precludes such comparisons with the Ontario populations reported in DiLeo et al. (2013).

We detected signatures of dispersal up to 1 km, which is similar to the average maximum range length measured in Eastern Massasaugas in the Bruce Peninsula, Ontario (Weatherhead & Prior, 1992), and within the range from several other studies (Szymanski, 1998). Our results indicate genetic spatial autocorrelation occurs at fine scales; therefore, the lack of strong observed genetic structuring suggests there is gene flow across the sampled area on BBI facilitated by suitable habitat acting as stepping stones (Crandall et al., 2012). Historically, Eastern Massasauga movement would have been aided by the decades of logging that occurred around the turn of the 20th century (Sanborn et al., 2012; Whitney, 1987) resulting in new corridors and open‐canopy habitat patches. While large‐scale logging has ceased, the current BBI landscape is more permeable to movement than more heavily impacted habitats found elsewhere in the species’ range, which is facilitated by aggregated habitat patches (Figure 2b). Contiguous swaths of forest are not ideal habitat, but gaps created by storms and tree falls provide thermoregulatory opportunities for snakes moving between habitat patches (Robillard & Johnson, 2015; author obs.).

While limited genetic structuring indicates an abundance of snakes in well‐connected habitats, we did find some evidence of barriers to movement. Genetic structure results must be carefully scrutinized in the presence of IBD (Perez et al., 2018), and the STRUCTURE genetic clusters seem to reflect an IBD (clinal) pattern rather than discrete spatial genetic clusters (Figures [Link], [Link]). Eastern Massasaugas from the southeastern corner of the island form the most homogenous cluster in the STRUCTURE results (Figures [Link], [Link]), and while this still fits an IBD pattern, the EEMS results support the idea that these snakes may be the most isolated on the island. Reduced effective migration was present in southeastern BBI (Figure 2c) coinciding with the two large, inland lakes and the area with the highest traffic volume on the island. DiLeo et al. (2013) showed open water deterred Eastern Massasauga gene flow in Ontario, and the BBI lakes likely represent a major barrier to gene flow as well. Eastern Massasaugas were detected in the small gap between the lakes so gene flow likely persists between the water barriers, albeit at a reduced rate. The combined effects of reduced migration from the northern side of the lakes and road mortality are likely contributing to lower effective genetic diversity (see heterozygosity estimates in Table A3) in southeastern BBI and limiting gene flow opportunities for the Eastern Massasaugas residing here.

### 4.2 Genetic diversity

The Massasauga population on Bois Blanc Island shows a level of genetic diversity that is on par with highly structured populations throughout the rest of the range. We might expect our island population to show reduced genetic diversity, relative to mainland sites, because of the combined effects of a potential founder effect (following colonization ~10,000 years ago), no migrants from the mainland (since the island was isolated ~4,500 years ago, Larsen, 1987), and genetic drift. However, our average observed heterozygosity (0.58) was equal to the range‐wide estimate reported from 19 sites by Chiucchi and Gibbs (0.58). This conversely indicates that highly structured populations elsewhere may be functional islands, experiencing similar levels of migration and drift as BBI. Many of these functional island populations are likely to be in more dire straits from demographic and genetic standpoints compared to BBI, occupying sites with less habitat that support fewer individuals. Sovic et al. (2019) detected genetic signatures for loss of diversity coinciding with the last two centuries from multiple populations in the southern part of the range. Different genetic markers used in DiLeo et al. (2013) make direct comparisons with the BBI results difficult but they reported allelic diversity estimates (10.3 and 10.9 alleles per locus) that are almost two times higher than ours (5.9 alleles per locus). The elevated genetic diversity seen in these Ontario populations may reflect their broad distribution and higher levels of connectivity compared to the southern part of the range.

### 4.3 Management implications

The findings suggest that Eastern Massasauga gene flow is possible when suitable habitat is abundant, and habitat patches are well connected. The short nearest neighbor distance (mean = 245 m) revealed by our habitat analysis illustrates the importance of minimizing spatial separation of patches, particularly in forested landscapes. Interpatch connectivity and suitable habitat area have not been formally assessed at other Eastern Massasauga sites. However, we expect similar habitat attributes (aggregated and in close proximity) are present in the Ontario landscapes sampled by DiLeo et al. (2013). This information can be used to promote greater connectivity among remaining Eastern Massasauga populations and to guide management efforts aimed at recolonizing areas that have experienced extirpation. Previous Eastern Massasauga recolonization studies have attempted to use translocations to recolonize previously occupied habitat and have found little success with high rates of mortality observed during overwintering (Harvey et al., 2014; King et al., 2004). Results from this study suggest that Eastern Massasaugas can (re)colonize new habitats, within reasonable distances (~1 km based on BBI genetic data), if the intervening landscape is traversable. Future studies should assess how best to restore Eastern Massasauga habitat, how fast and efficient Eastern Massasaugas are at recolonizing restored habitat, and what habitat types and landscape features may act as barriers to recolonization. Additionally, management of remaining Eastern Massasauga populations could be examined on a case by case basis. As we have shown, Eastern Massasauga populations in relatively undisturbed habitats behave very differently than their counterparts in heavily fragmented landscapes. Management could therefore account for these discrepancies by taking a broader landscape perspective.

Developing and employing management strategies to facilitate gene flow may aid Eastern Massasauga recovery. To prevent the further loss of genetic diversity and adaptive potential of Eastern Massasauga populations, increased gene flow among populations or genetic rescue via introductions or landscape management is likely required (Martin et al., 2021; Sovic et al., 2019). As genetic drift proceeds in isolated populations, it is estimated that in the next 100 years the genetic variation of Eastern Massasauga populations will decline by 20% (Sovic et al., 2019). This trend of declining genetic variation is further supported by the loss of 67% of rare alleles over a ten‐year time period from the single remaining Eastern Massasauga population in Illinois (Baker et al., 2018).

## 5 CONCLUSIONS

Spatial genetic structure studies on snakes are still rare despite a clear need for such information, given the global decline of reptiles (Gibbons et al., 2000; Todd et al., 2010). We used multiple analytic methods to assess fine‐scale genetic structuring for a species largely considered to be restricted to isolated populations due to habitat loss and limited mobility. We showed that in a landscape context with few anthropogenic disturbances, this species is capable of sufficient rates of movement and gene flow to prevent strong genetic structuring from arising at the same spatial scale at which highly structured populations occur in the southern part of the range. Restoring landscapes to resemble BBI may be difficult across much of the Eastern Massasauga's range, as this remote location has minimal human development pressures. However, our study is a demonstration that the migration ability of Eastern Massasaugas, coupled with habitat restoration that improves connectivity, could promote natural dispersal and colonization. This might be especially important for conservation in those parts of the species’ range with more fragmented landscapes than BBI. There have already been genetic consequences for several of the habitat island populations in the southern part of the Eastern Massasauga's range (Martin et al., 2021; Sovic et al., 2019) and expanding the occupied area might require population augmentation measures, with considerations for local adaptation, to revitalize the gene pool in those areas to provide insurance from future stochastic events that might tend to diminish genetic variation.

## CONFLICT OF INTEREST

The authors declare no conflict of interest.

## AUTHORS’ CONTRIBUTIONS

Nathan Kudla (cofirst author): Conceptualization; investigation; methodology; and writing—original draft preparation. Eric McCluskey (cofirst author): Conceptualization; funding acquisition; methodology; and writing—original draft preparation. Vijay Lulla: Software; visualization; and writing—review and editing. Ralph Grundel: Conceptualization; funding acquisition; writing—review and editing. Jennifer Moore: Conceptualization; funding acquisition; methodology; supervision; and writing—review and editing.

## Supporting information

Supplementary Material

Supplementary Material

Supplementary Material

Supplementary Material

Supplementary Material

Supplementary Material

Supplementary Material

Supplementary Material

## Data Availability

Genotype data for all sampled individuals can be accessed at https://doi.org/10.5066/P9HJW59U. We are unable to provide georeferenced genetic data used for most of the analyses in this manuscript due to the threats both illegal collection and indiscriminate killing pose to Eastern Massasauga populations on Bois Blanc Island and across their range. The Michigan Department of Natural Resources and U. S. Fish & Wildlife Service have both expressed concern over the release of any Eastern Massasauga location data. ​
