# Supplementary figures and images for: Intact landscape promotes gene flow and low genetic structuring in the threatened Eastern Massasauga Rattlesnake
Source: Ecol Evol. 2021 May 2;11(11):6276–88. doi: 10.1002/ece3.7480 (PMC8207425; doi:10.1002/ece3.7480)

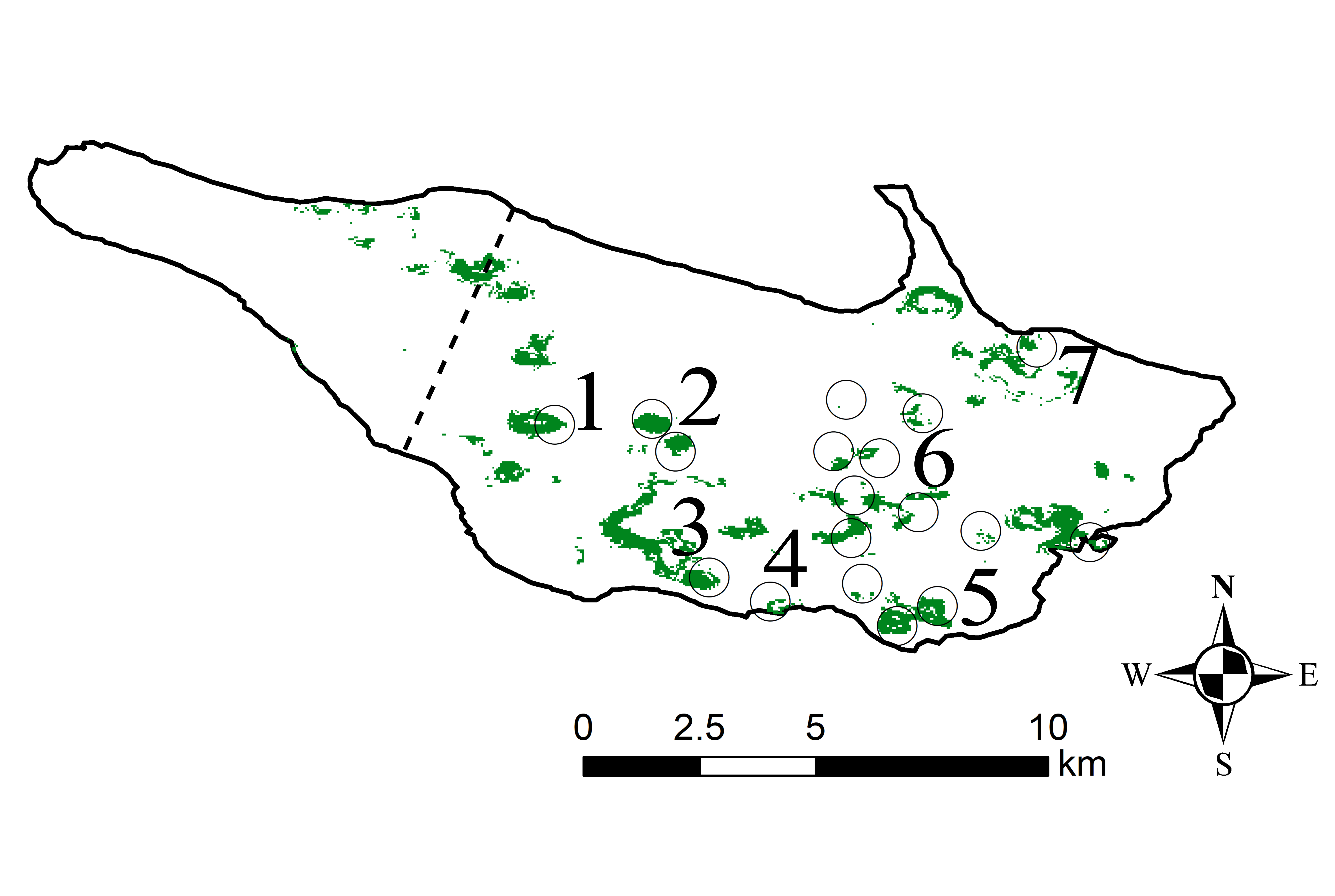

Supplement: Supplementary file 1 — Supplementary Material [file ECE3-11-6276-s003.png]

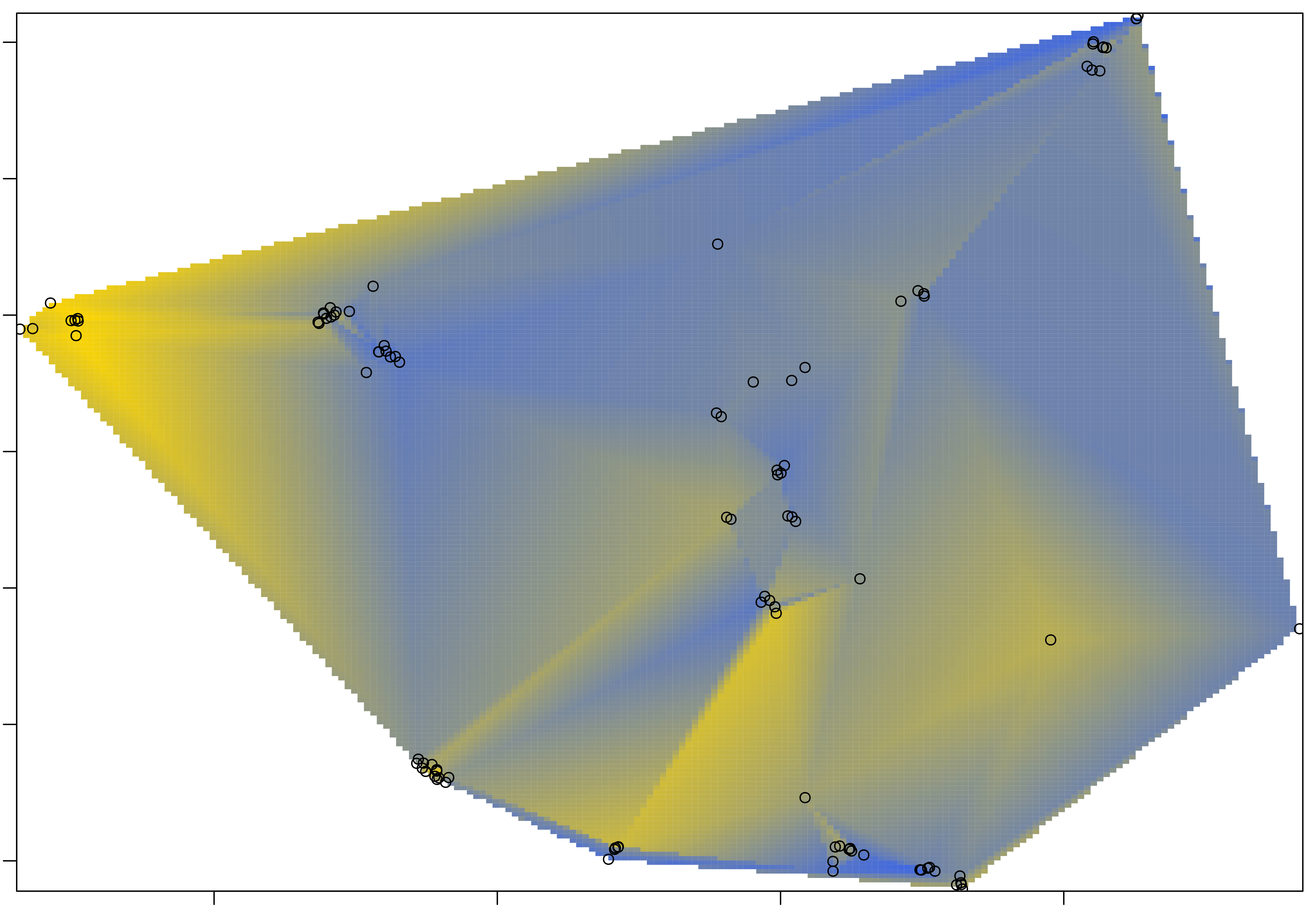

Supplement: Supplementary file 2 — Supplementary Material [file ECE3-11-6276-s001.tif]

Delta K

25  
20  
15  
10  
5  
0

1 2 3 4 5 6 7 8 9 10 11 12 13 14 15

K

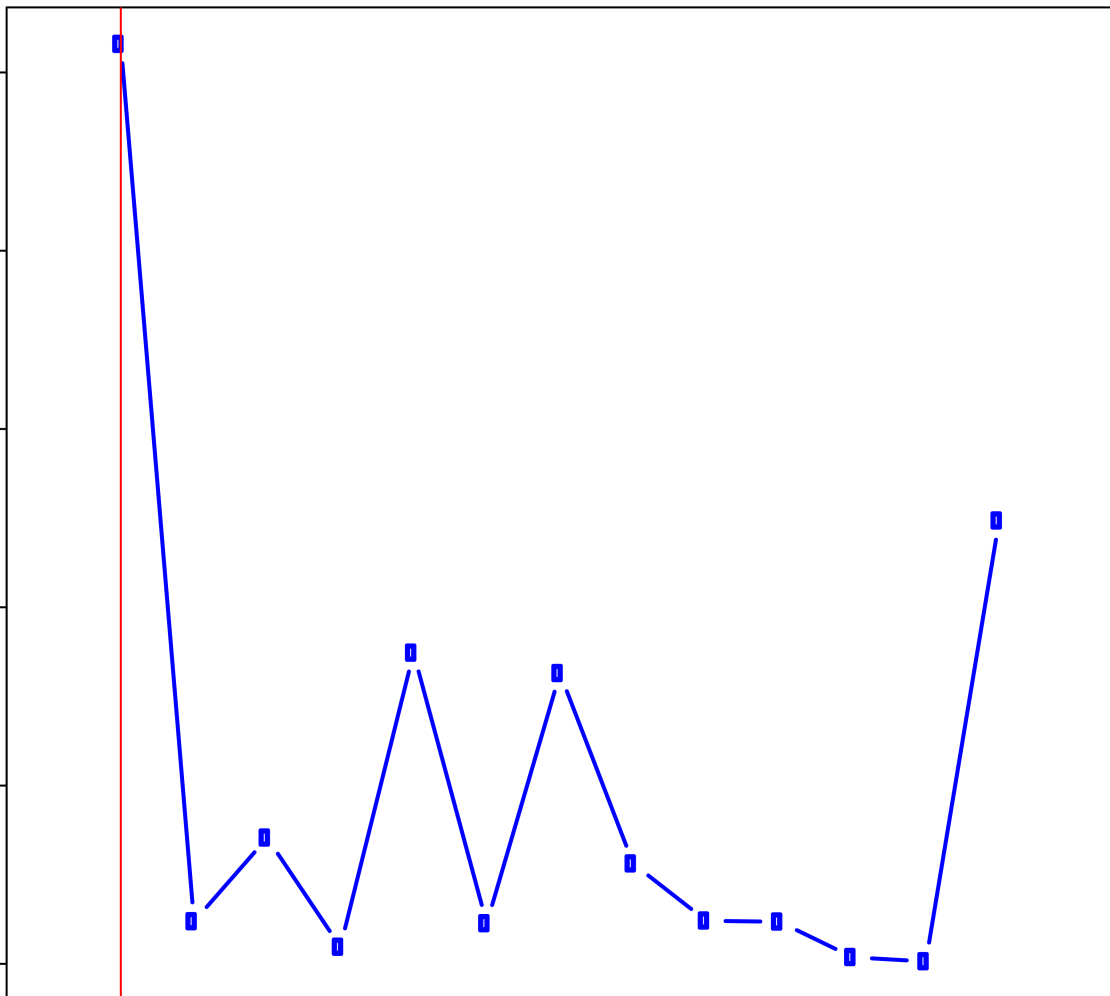

Supplement: Supplementary file 3 — Supplementary Material [file ECE3-11-6276-s007.pdf]

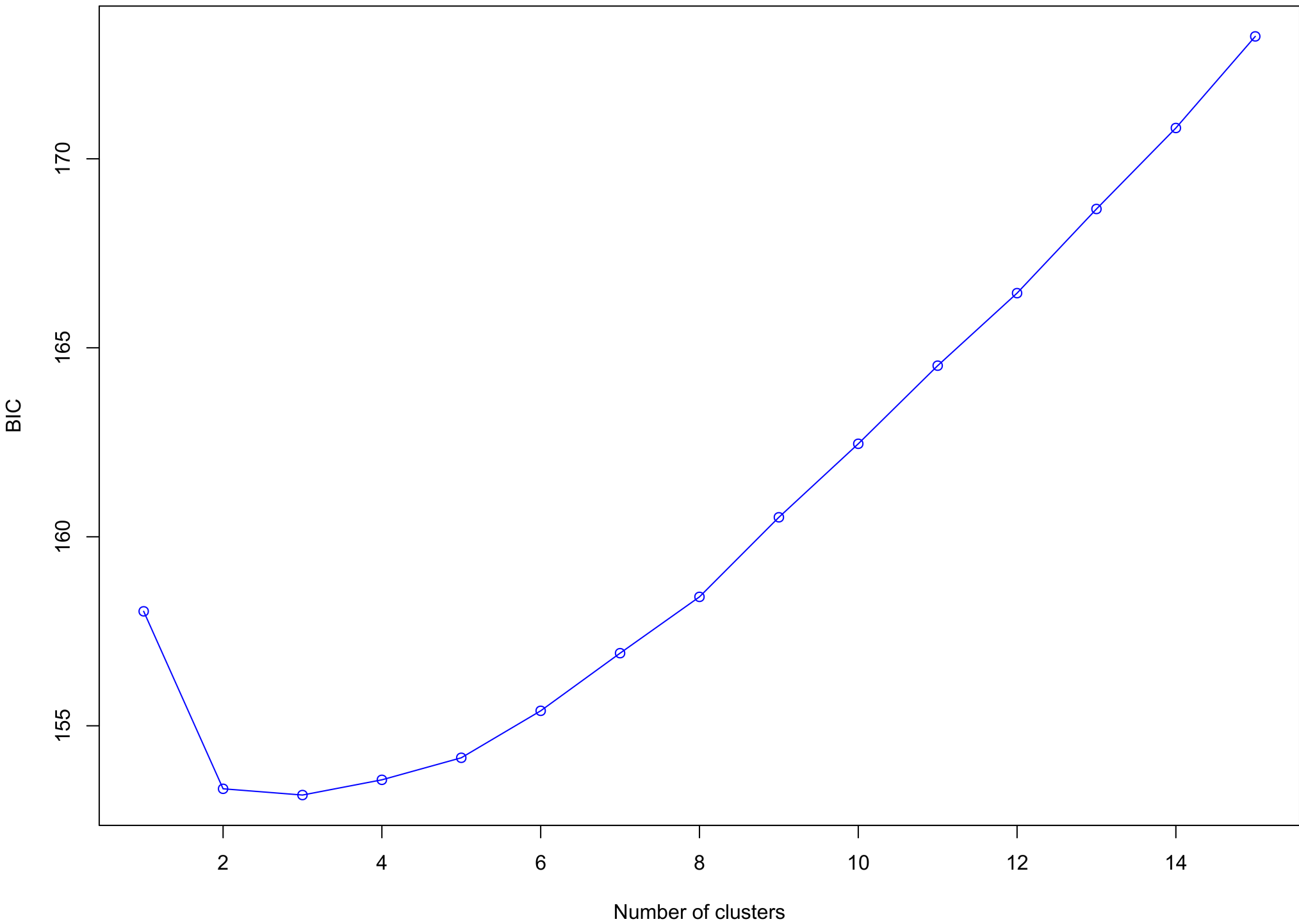

Supplement: Supplementary file 4 — Supplementary Material [file ECE3-11-6276-s004.pdf]

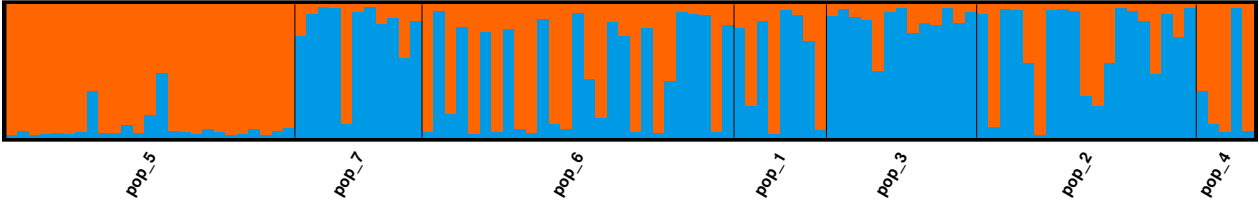

Supplement: Supplementary file 5 — Supplementary Material [file ECE3-11-6276-s008.pdf]

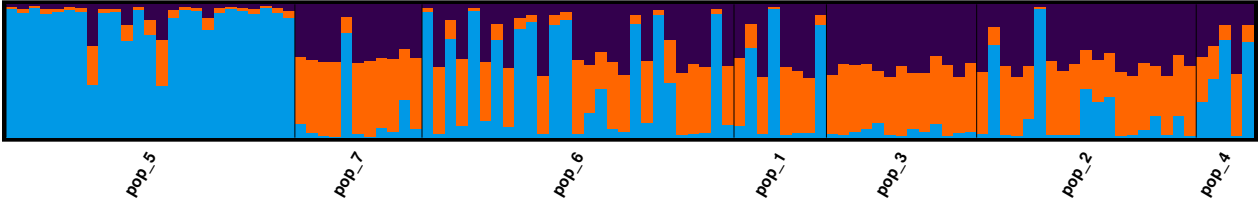

Supplement: Supplementary file 6 — Supplementary Material [file ECE3-11-6276-s002.pdf]
