# Supplementary material for: Intact landscape promotes gene flow and low genetic structuring in the threatened Eastern Massasauga Rattlesnake
Source: Ecol Evol. 2021 May 2;11(11):6276–88. doi: 10.1002/ece3.7480 (PMC8207425; doi:10.1002/ece3.7480)
Supplement: Supplementary file 8 — Supplementary Material [file ECE3-11-6276-s005.docx]

Table 1. Genetic diversity statistics for 19 Eastern Massasauga for 15 microsatellite loci from the western high genetic diversity region on Bois Blanc Island identified by EEMS (estimated effective migration surfaces) and organized by microsatellite locus including number of alleles per locus (N_a_), allelic richness (AR), observed heterozygosity (H_o_), and expected heterozygosity (H_e_).

| Locus | N_a_ | AR | H_o_ | H_e_ |
| --- | --- | --- | --- | --- |
| *Scu201* | 3 | 2.44 | 0.26 | 0.24 |
| *Scu202* | 5 | 4.67 | 0.63 | 0.72 |
| *Scu203* | 3 | 2.99 | 0.63 | 0.61 |
| *Scu204* | 4 | 3.75 | 0.68 | 0.69 |
| *Scu205* | 6 | 4.87 | 0.74 | 0.73 |
| *Scu206* | 2 | 2.00 | 0.32 | 0.40 |
| *Scu208* | 3 | 2.25 | 0.16 | 0.15 |
| *Scu210* | 6 | 5.32 | 0.79 | 0.79 |
| *Scu211* | 7 | 5.86 | 0.84 | 0.78 |
| *Scu212* | 4 | 3.70 | 0.58 | 0.56 |
| *Scu213* | 4 | 3.49 | 0.47 | 0.63 |
| *Scu214* | 4 | 2.75 | 0.21 | 0.20 |
| *Scu215* | 5 | 4.67 | 0.68 | 0.74 |
| *Scu216* | 7 | 5.81 | 0.79 | 0.79 |
| *Scu217* | 5 | 4.67 | 0.63 | 0.72 |
| **Average** | **4.53** | **3.95** | **0.56** | **0.58** |

Table 2. Genetic diversity statistics for 11 Eastern Massasauga for 15 microsatellite loci from the eastern high genetic diversity region on Bois Blanc Island identified by EEMS (estimated effective migration surfaces) and organized by microsatellite locus including number of alleles per locus (N_a_), allelic richness (AR), observed heterozygosity (H_o_), and expected heterozygosity (H_e_).

| Locus | N_a_ | AR | H_o_ | H_e_ |
| --- | --- | --- | --- | --- |
| *Scu201* | 3 | 2.99 | 0.91 | 0.63 |
| *Scu202* | 4 | 3.94 | 0.82 | 0.71 |
| *Scu203* | 3 | 3.00 | 0.82 | 0.67 |
| *Scu204* | 3 | 2.76 | 0.36 | 0.56 |
| *Scu205* | 6 | 5.45 | 0.82 | 0.77 |
| *Scu206* | 2 | 1.99 | 0.27 | 0.25 |
| *Scu208* | 2 | 1.99 | 0.27 | 0.25 |
| *Scu210* | 5 | 4.70 | 0.73 | 0.75 |
| *Scu211* | 6 | 5.45 | 0.73 | 0.74 |
| *Scu212* | 4 | 3.94 | 0.82 | 0.73 |
| *Scu213* | 3 | 3.00 | 0.82 | 0.69 |
| *Scu214* | 3 | 2.89 | 0.27 | 0.33 |
| *Scu215* | 4 | 3.94 | 0.64 | 0.70 |
| *Scu216* | 5 | 4.65 | 0.64 | 0.71 |
| *Scu217* | 4 | 3.94 | 0.82 | 0.71 |
| **Average** | **3.80** | **3.64** | **0.65** | **0.61** |

Table 3. Genetic diversity statistics for 46 Eastern Massasauga for 15 microsatellite loci from the low genetic diversity region on Bois Blanc Island identified by EEMS (estimated effective migration surfaces) and organized by microsatellite locus including number of alleles per locus (N_a_), allelic richness (AR), observed heterozygosity (H_o_), and expected heterozygosity (H_e_).

| Locus | N_a_ | AR | H_o_ | H_e_ |
| --- | --- | --- | --- | --- |
| *Scu201* | 4 | 3.19 | 0.37 | 0.36 |
| *Scu202* | 6 | 4.48 | 0.54 | 0.51 |
| *Scu203* | 3 | 2.99 | 0.52 | 0.58 |
| *Scu204* | 4 | 3.94 | 0.57 | 0.72 |
| *Scu205* | 8 | 4.87 | 0.72 | 0.72 |
| *Scu206* | 2 | 2.00 | 0.43 | 0.39 |
| *Scu208* | 3 | 2.02 | 0.15 | 0.14 |
| *Scu210* | 7 | 5.87 | 0.83 | 0.78 |
| *Scu211* | 7 | 5.73 | 0.74 | 0.82 |
| *Scu212* | 4 | 3.81 | 0.57 | 0.59 |
| *Scu213* | 5 | 4.21 | 0.54 | 0.56 |
| *Scu214* | 4 | 2.60 | 0.20 | 0.20 |
| *Scu215* | 5 | 4.71 | 0.78 | 0.76 |
| *Scu216* | 11 | 6.39 | 0.78 | 0.79 |
| *Scu217* | 7 | 4.32 | 0.48 | 0.47 |
| **Average** | **5.33** | **4.08** | **0.55** | **0.56** |

Table 4A-C. Evaluation metrics for 10 independent model runs from (A) ANN, (B) Maxent, and (C) ANN/Maxent (EF) ensemble model types including sensitivity, specificity, area under the curve (AUC), true skill statistic (TSS), Somers’ D, and Boyce Index.

A)

| Model | Sensitivity | Specificity | AUC | TSS | SomersD | Boyce |
| --- | --- | --- | --- | --- | --- | --- |
| RUN1_ANN | 1.00 | 0.84 | 0.93 | 0.84 | 0.87 | 0.77 |
| RUN2_ANN | 0.83 | 0.81 | 0.88 | 0.65 | 0.75 | 0.88 |
| RUN3_ANN | 0.83 | 0.81 | 0.83 | 0.64 | 0.66 | 0.82 |
| RUN4_ANN | 0.83 | 0.87 | 0.90 | 0.70 | 0.80 | 0.92 |
| RUN5_ANN | 0.83 | 0.95 | 0.93 | 0.78 | 0.87 | 0.81 |
| RUN6_ANN | 0.67 | 0.91 | 0.84 | 0.57 | 0.68 | 0.84 |
| RUN7_ANN | 1.00 | 0.62 | 0.86 | 0.62 | 0.71 | 0.67 |
| RUN8_ANN | 1.00 | 0.58 | 0.86 | 0.58 | 0.71 | 0.84 |
| RUN9_ANN | 1.00 | 0.81 | 0.92 | 0.81 | 0.83 | 0.68 |
| RUN10_ANN | 1.00 | 0.79 | 0.89 | 0.79 | 0.79 | 0.71 |
| **Average** | **0.90** | **0.80** | **0.88** | **0.70** | **0.77** | **0.80** |

B)

| Model | Sensitivity | Specificity | AUC | TSS | SomersD | Boyce |
| --- | --- | --- | --- | --- | --- | --- |
| RUN1_MAXENT | 1.00 | 0.82 | 0.94 | 0.82 | 0.89 | 0.67 |
| RUN2_MAXENT | 1.00 | 0.64 | 0.87 | 0.64 | 0.75 | 0.90 |
| RUN3_MAXENT | 0.83 | 0.83 | 0.88 | 0.66 | 0.75 | 0.93 |
| RUN4_MAXENT | 0.83 | 0.90 | 0.90 | 0.73 | 0.81 | 0.93 |
| RUN5_MAXENT | 0.83 | 0.92 | 0.93 | 0.75 | 0.87 | 0.92 |
| RUN6_MAXENT | 0.83 | 0.86 | 0.86 | 0.70 | 0.72 | 0.81 |
| RUN7_MAXENT | 1.00 | 0.64 | 0.84 | 0.64 | 0.69 | 0.86 |
| RUN8_MAXENT | 1.00 | 0.64 | 0.86 | 0.64 | 0.73 | 0.79 |
| RUN9_MAXENT | 1.00 | 0.83 | 0.93 | 0.83 | 0.86 | 0.81 |
| RUN10_MAXENT | 1.00 | 0.84 | 0.92 | 0.84 | 0.84 | 0.70 |
| **Average** | **0.93** | **0.79** | **0.89** | **0.72** | **0.79** | **0.83** |

C)

| Model | Sensitivity | Specificity | AUC | TSS | SomersD | Boyce |
| --- | --- | --- | --- | --- | --- | --- |
| RUN1_EF | 1.00 | 0.83 | 0.94 | 0.83 | 0.88 | 0.82 |
| RUN2_EF | 0.83 | 0.78 | 0.88 | 0.61 | 0.76 | 0.91 |
| RUN3_EF | 0.83 | 0.85 | 0.86 | 0.68 | 0.71 | 0.55 |
| RUN4_EF | 0.83 | 0.90 | 0.90 | 0.73 | 0.81 | 0.91 |
| RUN5_EF | 0.83 | 0.94 | 0.94 | 0.77 | 0.87 | 0.96 |
| RUN6_EF | 0.83 | 0.81 | 0.85 | 0.65 | 0.71 | 0.76 |
| RUN7_EF | 1.00 | 0.62 | 0.85 | 0.62 | 0.70 | 0.79 |
| RUN8_EF | 1.00 | 0.62 | 0.86 | 0.62 | 0.72 | 0.83 |
| RUN9_EF | 1.00 | 0.85 | 0.93 | 0.85 | 0.85 | 0.78 |
| RUN10_EF | 1.00 | 0.81 | 0.91 | 0.81 | 0.83 | 0.75 |
| **Average** | **0.92** | **0.80** | **0.89** | **0.72** | **0.78** | **0.80** |

Figure 1. Seven sampling areas (some consist of several adjacent sites indicated by circles) across Bois Blanc Island selected as predefined populations for the implementation of the Puechmaille (2016) genetic clustering method using StructureSelector.

Figure 2. Genetic clines across the Eastern Massasauga sampling areas on Bois Blanc Island from the interpolation of lagged principal scores. The sPCA showed no significant spatial structure. Coordinates were intentionally removed to deter poachers.

Figure 3. Selection of the optimal number of genetic clusters (K=2) for 102 Eastern Massasauga samples on Bois Blanc Island using the Delta K Evanno method on STRUCTURE output.

Figure 4. Selection of three genetic clusters (K=3) for 102 Eastern Massasauga samples on Bois Blanc Island using DAPC method in the R package ADEGENET. The y-axis shows the Bayesian information criterion (BIC) values.

Figure 5. Structure plot showing two genetic clusters (K=2) across seven Eastern Massasauga sampling areas on Bois Blanc Island.

Figure 6. Structure plot showing three genetic clusters (K=3) across seven Eastern Massasauga sampling areas on Bois Blanc Island.
